# Methodological considerations in the ADAPT-1 ovarian stimulation study

**DOI:** 10.1093/humrep/deag120

**Published:** 2026-07-23

**Authors:** Susana Montenegro, Christoph Helwig

**Affiliations:** Merck Healthcare KGaA, Darmstadt, Germany; Merck Healthcare KGaA, Darmstadt, Germany

Dear Editors,

We read with interest the article by Bernabeu *et al.* (2025) describing the ADAPT-1 trial comparing recombinant human follicle-stimulating hormone (r-hFSH) delta with alfa. While the authors should be commended for adding clinical data on ovarian stimulation, several pharmacological and methodological issues temper the interpretation of its results. We outline considerations that are critical for readers.

## Lack of bioequivalence and unreliable IU-to-µg conversion

Although follitropin delta and alfa share the same FSH DNA and amino acid sequence, their glycosylation profiles differ substantially: follitropin delta contains both α2,3- and α2,6-linked sialic acids and more tri-/tetra-antennary structures, whereas follitropin alfa has only α2,3-linked sialic acids (Håkan and Grundemar, 2014). These glycosylation differences are not minor; they alter pharmacokinetics and receptor interactions, leading to higher exposure (AUC) and reduced clearance for follitropin delta in preclinical models (Arce *et al.*, 2014; Monica *et al.*, 2023), and are expected to influence both bioassay readouts and clinical efficacy in humans. Precisely because the rat Steelman–Pohley bioassay underestimates follitropin delta activity in humans, the follitropin delta SmPC (Summary of Product Characteristics) mandates dosing in micrograms and explicitly states that this mass-based dose cannot be extrapolated to other gonadotropins (SmPC, 2021). Despite this, the ADAPT-1 trial assumed a conversion factor of 15 µg ≈ 225 IU as a working approximation. However, this empiric factor is not scientifically justified as a general equivalence metric, does not establish bioequivalence between follitropin delta and alfa, and cannot be validly used across different dosing regimens or patient populations (Bernabeu *et al.*, 2025).

## Off-label conventional dosing deviates from the authorized delta algorithm

The follitropin delta’s SmPC restricts its first-cycle dosing to ≤12 µg/day and requires an Anti-Müllerian Hormone (AMH) and body weight-based algorithm, use with no intra-cycle dose adjustments (SmPC, 2021). In ADAPT-1 follitropin delta was administered at 15 µg/day, exceeding the authorized limit and bypassing the required algorithm, and follitropin alfa at 225 IU/day, both fixed for 4 days, with adjustments from Day 5 or 6 (Bernabeu *et al.*, 2025). Consequently, ADAPT-1 neither validates the approved follitropin delta algorithm nor justifies abandoning it, and extrapolating these results to routine practice risks misrepresenting follitropin delta’s risk–benefit profile outside its labeled use.

## Safety findings support equivalence of ovarian hyperstimulation syndrome (OHSS) risk under fix dosing regimens

ADAPT-1 delivers a crucial message: when follitropin delta and alfa are used within comparable dosing paradigms, their OHSS risk profiles converge. In ADAPT‑1, early OHSS rates and the use of preventive interventions were virtually identical between follitropin delta and follitropin alfa (2.5 vs 3.0% early OHSS; 15 vs 15% preventive measures), with no cycles canceled for excessive response (Bernabeu *et al.*, 2025). These findings suggest that previously reported OHSS reductions with follitropin delta mainly reflect the limitations of earlier study designs (Gouveia *et al.*, 2022) comparing individualized, algorithm-based dosing of follitropin delta with fixed-dose follitropin alfa—rather than any intrinsic molecular safety advantage of follitropin delta over other r-hFSH preparations. Accurate scientific communication should reflect this distinction to ensure informed, guideline-consistent clinical decisions.

## Descriptive design cannot support proof of ‘equivalence’

ADAPT-1 was planned as a descriptive comparative analysis without inferential statistics and with no formal hypothesis testing; no prospectively defined equivalence or non-inferiority margins, hierarchical hypotheses, or multiplicity controls were specified. Regulatory guidance requires *a priori* justification of the non-inferiority margin and demonstration that the 95% CI lies entirely above that margin (E9, 1998; E10, 2000). The reported 95% CI for the oocyte difference (−1.44 to +1.44) is narrow, but it cannot establish therapeutic equivalence or interchangeability without an explicit intention to demonstrate equivalence or non-inferiority.

## Additional design and analytical limitations

Metaphase II oocyte counts were not reported separately despite being captured for all 300 cycles. Dose adjustments and multiple pregnancy data were also not reported, yet potential bias from these exclusions was not discussed. Additionally, treating total microgram dose equivalently across products overlooks the fact that 1 µg alfa ≠ 1 µg delta (Gouveia *et al.*, 2022; Monica *et al.*, 2023; Montenegro *et al.*, 2025).

Collectively, these disputes indicate that the ADAPT-1 trial provides useful descriptive information on one fixed-dose comparison in normoresponders but cannot establish therapeutic equivalence, interchangeability, or a valid IU-to-µg conversion. Future studies should employ each r-hFSH within its authorized dosing regimen, account for the implications of follitropin delta’s glycosylation profile, and adopt prespecified equivalence or non-inferiority frameworks compliant with International Council for Harmonization (ICH) standards (E9, 1998; E10, 2000).
